# Plasticity, signaling, and metabolic rewiring in melanoma persister cells

**DOI:** 10.1038/s42003-026-10143-w

**Published:** 2026-04-28

**Authors:** Shayne Sensenbach, Han G. Ngo, Mehmet A. Orman

**Affiliations:** 1 Department of Chemical and Biomolecular Engineering, University of Houston, Houston, TX USA; 2 Department of Biomedical Engineering, University of Wisconsin-Madison, Madison, WI USA

**Keywords:** Melanoma, Cancer therapy

## Abstract

Despite advances in therapy, survival rates for metastatic melanoma remain low. Drug-tolerant persister cells (persisters) can facilitate cancer recurrence, yet their characteristics and vulnerabilities differ across cancers and even within melanoma, depending on mutational context and treatment strategy. Both preexisting phenotypic traits and drug-induced adaptations contribute to persister formation, linked by “primed” persisters that exhibit intermediate states observed in multiple studies. Compared with parental melanoma cells and other phenotypic variants such as melanoma stem cells or senescent cells, persisters show altered and reversible differentiation programs, distinct metabolic adaptations, and rewired signaling networks, all affected by the tumor microenvironment, but remain poorly understood. This review focuses on melanoma persisters, highlighting advances in understanding their origins, signaling plasticity, metabolic rewiring, and therapeutic vulnerabilities. By identifying gaps, this review further provides much-needed recommendations for future research and outlines priorities for advancing persister-directed interventions toward clinical translation.

## Abstract (web summary)

This review summarizes the origins, plasticity, signaling dynamics, and metabolic rewiring of melanoma persister cells, highlighting their role in therapy resistance and recurrence, and identifying vulnerabilities and future directions.

## Introduction

Melanoma is a pressing health concern in the United States and globally. The American Cancer Society projects 104,960 melanoma diagnoses in the United States in 2025, resulting in 8430 deaths^1^. Incidence rates for melanoma have risen over the past several decades^2^, and recurrence rates above 40% have been reported for metastatic melanoma^3^. Most melanoma tumors harbor oncogenic mutations in the rapidly accelerated fibrosarcoma B-type (*BRAF*) gene, specifically *BRAF* V600E mutations, which drive continuous, uncontrolled proliferation^4,5^. Therefore, targeted therapeutics that block this oncogenic pathway, including BRAF and mitogen-activated protein kinase (MEK) inhibitors (BRAFi and MEKi, respectively), are widely used to treat metastatic melanoma^6^. Prior to the discovery of these modern therapeutics, traditional chemotherapeutics were the only option, and these drugs are ineffective in melanoma^6^. While BRAFi and MEKi have drastically improved response rates in melanoma, recurrence and the development of drug resistance mechanisms are nearly certain for advanced cases^6–8^.

Persistence, a transient form of drug tolerance, is an important phenomenon related to cancer recurrence and treatment failure in general. “Persisters” were originally discovered in bacterial cultures in the 1940s, and they were characterized as a rare, slow- or non-growing sub-population of cells that tolerate antibiotic treatment^9,10^. More recently, the slow- or non-growing, drug-tolerant phenotype was also discovered in sub-populations of cancer cells, and these cells are known as cancer persisters^11–13^. Clinically, these cancer persisters are thought to contribute to minimal residual disease (MRD) and recurrence^11–14^, and the contribution of persisters to these phenomena has been demonstrated in animal experiments^15^. Cancer persisters have been discovered in many cancer types upon treatment with various anticancer agents, including traditional chemotherapeutics and targeted therapeutics^11–13^. However, the characteristics of cancer persisters vary depending on the cell type, mutational status, and the treatment strategy used. Additionally, persisters are especially important in melanoma treatment, due to the high rates of recurrence in melanoma^3^, as well as the high levels of genetic and epigenetic diversity shown in melanomas compared to other cancers^16–18^.

Several recent review articles have focused broadly on melanoma, summarizing the treatment methods and the challenges posed by the disease^6,19–21^. Cancer persisters in general have also been the topic of multiple reviews^22–28^. While broad overviews are necessary, in-depth articles focusing on persisters of specific cancer types or their specific characteristics would be useful to researchers in these areas. For example, Rodriguez et al. reviewed the importance of iron and ferroptosis in persisters^29^, and Cabanos and Hata focused specifically on cancer persisters which tolerate targeted therapeutics^30^. However, to date, reviews specifically focusing on melanoma persisters remain limited. Given the extensive research on melanoma persisters, our aim in this review is to highlight the known characteristics of melanoma persisters specifically and recommend key areas to focus future research.

## Main

### Treatment strategies and persister formation

Traditionally, metastatic melanoma has been treated with chemotherapeutics, such as dacarbazine and temozolomide, resulting in dismal response and survival rates^6^. While treatment outcomes, including survival rates, have shown significant improvement when patients are treated with BRAFi and MEKi^21^, overall five-year survival rates have only climbed to 35% for distant metastatic cases^1^. Immunotherapy is another promising treatment strategy that has recently been implemented for melanoma^6^; however, research on melanoma persisters in the context of immunotherapy is limited (see the “Future directions” section for more details). Therefore, most of the articles discussed in this review focus on treatments with chemotherapeutics or targeted therapeutics.

**Fig. 1:**
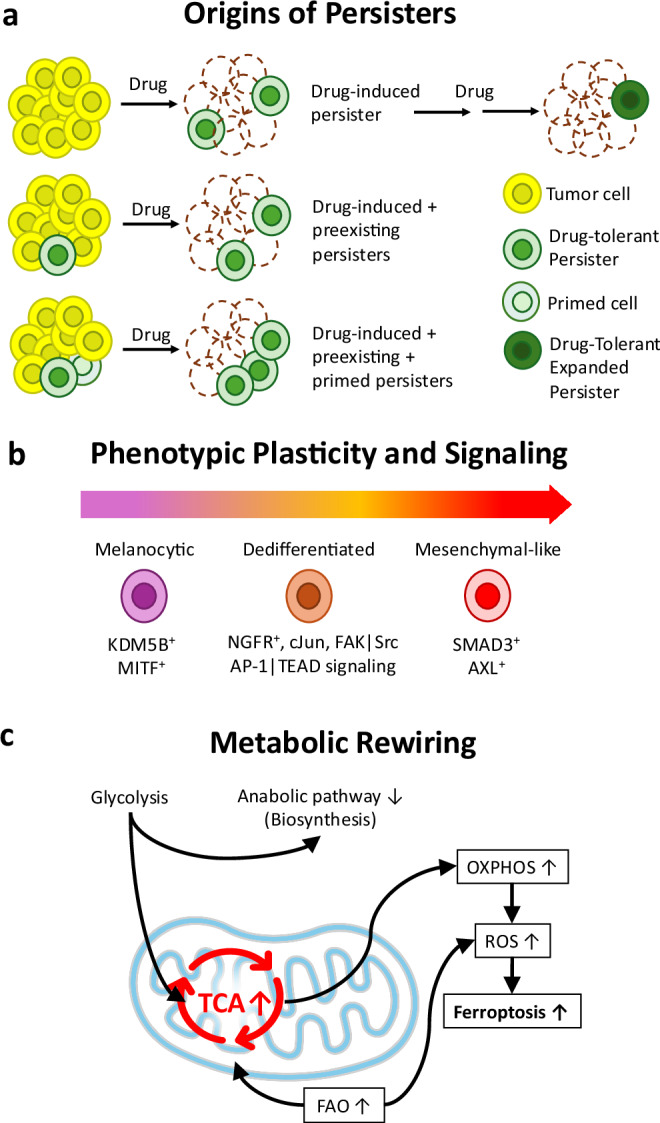
Overview of melanoma persisters: origins, plasticity, and metabolic rewiring. **a** Origins of persisters. Melanoma persisters can emerge as rare preexisting cells with intrinsic survival traits such as receptor tyrosine kinase upregulation, as drug-induced persisters formed under treatment stress through epigenetic and metabolic reprogramming, or as primed persisters in intermediate states that predispose them to tolerance. These origins are not mutually exclusive and likely represent a continuum of overlapping processes. A prolonged treatment phase can facilitate the emergence of drug-tolerant expanded persisters (DTEPs) under sustained therapeutic pressure. **b** Phenotypic plasticity and signaling. Persisters adopt diverse and reversible states, including melanocytic-like (KDM5B⁺, MITF⁺), mesenchymal-like (SMAD3⁺, AXL⁺, ferroptosis-prone), and dedifferentiated NGFR⁺ cells (SOX10 loss, cJun, FAK/Src/AP-1/TEAD signaling). These transitions reflect transcriptional, epigenetic, and translational remodeling. **c** Metabolic rewiring. Persister survival involves increased fatty acid oxidation (FAO), enhanced tricarboxylic acid (TCA) cycle activity, and elevated oxidative phosphorylation (OXPHOS), driving higher reactive oxygen species (ROS) levels and ferroptosis sensitivity, while glycolysis and anabolic metabolism are reduced. Icon reproduced from Microsoft PowerPoint (Microsoft 365), used under license.

Both traditional chemotherapeutics and targeted therapeutics can halt cell proliferation and induce cell death in a majority of melanoma cells, but both can leave behind troublesome persister populations. Regarding melanoma persister cell formation, two intertwined hypotheses are at play: (1) preexisting persisters may survive treatment due to certain patterns of gene expression, metabolic activity, signaling pathway activity, or differentiation state, or (2) these pro-survival profiles may be induced by the treatments themselves (Fig. 1a). An example of a preexisting survival advantage is the variable expression of receptor tyrosine kinases (RTKs, e.g., EGFR)^31,32^, which act upstream of BRAF, and can reactivate the oncogenic signal during targeted inhibition. RTK expression and activity levels were found to be higher in persisters than in drug-sensitive cells, and this difference was crucial to the persisters’ treatment evasion^31,32^. As for the drug-induced hypothesis, our research group has shown that melanoma cells transiently shift to a persister state, characterized by an altered metabolic profile including increased mitochondrial respiration, during treatment with various traditional chemotherapeutics^33^. The concept of drug-induced persistence is also supported by the dependence of persisters on epigenetic modifiers, which has been shown in several articles^11,34–37^. We think these two hypotheses are not mutually exclusive; in fact, they may act in concert to determine whether a cell adopts a persister phenotype. For example, the existence of “primed” persister cells, which are poised to adapt and enter a drug-tolerant state upon drug exposure, has been reported in melanoma^38,39^, serving as a connection between preexisting and drug-induced persisters. Clearly, the types of persisters present and how they form are dependent on the genomic and epigenetic profiles of the tumor cells, as well as the treatment strategies used, and other factors such as intratumoral phenotypic heterogeneity and plasticity.

### Crucial differentiation states and signaling pathways in melanoma persisters

Many articles throughout cancer persister research compare differentiation states and signaling pathway activity levels in persisters vs. the drug naïve parental cells. In fact, in a pioneering study in the field of cancer persister research, Sharma et al. found that PC9 lung cancer persisters survived EGFR inhibition through histone demethylase KDM5A-dependent chromatin remodeling^11^. This epigenetic shift led to enhanced IGF-1R signaling, enabling persister survival^11^. Interestingly, another histone demethylase, KDM5B, is highly expressed in melanoma persisters surviving BRAFi and MEKi treatments^35,40^. The persisters with high KDM5B expression exhibit slow growth, low mesenchymal gene expression, and high melanocytic differentiation^35^. However, many other articles have seemingly contradicted these findings, strengthening the idea that the characteristics of persister cell populations vary and depend on numerous factors. Specifically, melanoma persisters surviving BRAFi and MEKi have been shown to exhibit increased mesenchymal gene expression^12,41,42^, and to shift into a dedifferentiated state with high NGFR expression^34,43,44^ (Fig. 1b).

Epithelial-to-mesenchymal transition (EMT) has long been linked to drug resistance^45^, so it is not surprising that mesenchymal characteristics are frequently found in persisters. Indeed, persisters derived from several cancer types treated with various drugs, including melanoma cells treated with BRAFi and MEKi, exhibit upregulated mesenchymal markers^12^. The mesenchymal phenotype has also been connected with SMAD3 activity in BRAFi persisters, and SMAD3 signaling led to the acquisition of drug resistance via activation of known drug-resistance genes, such as EGFR and AXL^41^. The same research group also discovered Aryl hydrocarbon Receptor (AhR) activity in persisters^46^, and this signaling pathway was also shown to be important to the SMAD3 active persisters^41^. Interestingly, in *NRAS*-mutant melanoma cells without *BRAF* mutations, mesenchymal-like cells were shown to survive MEKi treatment^42^.

Regarding the observed expression of NGFR, this receptor is associated with neural crest stem cells, explaining the low levels of melanocytic differentiation in these persisters^34,43^. Key pro-survival signaling pathway changes in the dedifferentiated persisters involve activation of c-Jun, FAK, and Src, as well as ECM remodeling (Fig. 1b)^34^; FAK also activates the AKT pathway, aiding the survival of persisters^43^. SOX10 inactivation has been shown as a key event in melanoma persister dedifferentiation upon BRAFi treatment, facilitating reprogramming and resistance acquisition through activation of AP-1 and TEAD signaling^39^. In this case, highly variable transcriptional profiles led to rare cells with high expression of drug resistance markers, and these rare cells were primed to survive and reprogram upon treatment^39^. Aside from NGFR, MITF is another protein that is important for melanocyte differentiation, and a zebrafish model produced melanoma persisters in vivo via temperature-controlled MITF expression^15^. Inactivation of MITF led to dedifferentiation and persister formation, and these persisters regrew into recurrent tumors upon MITF reactivation^15^. Therefore, these findings show a similar trend to the high expression of NGFR commonly observed in melanoma persisters treated with single or combination BRAFi and MEKi.

The expression and signaling activity levels of RTKs are also important to melanoma persisters treated with BRAFi and MEKi^31,32^. In fact, under drug treatment, these persisters were shown to rewire their MAPK signaling into a receptor-driven format, as opposed to being driven by oncogenic signaling^31^. This adaptation led to a growth signal that was much more drug-resistant than in the parental cells^31^. Persisters were also shown to have a stronger and more sustained activation of RTK signaling upon stimulation compared to untreated cells^32^.

Interestingly, mRNA modifications and translation remodeling have been identified in melanoma BRAFi and MEKi persisters^36^. While the persisters had an overall reduction in protein synthesis compared to parental cells, they translated certain pro-survival mRNAs, including chromatin remodelers and stress-responsive kinases, at increased efficiencies^36^. These results suggest that the adaptation of melanoma persisters to drug treatments may begin with transcriptional and translational changes^36^, which can then lead to additional phenotypes discussed here, such as chromatin remodeling, shifts in gene expression, and signaling pathway modulation.

Aside from those which have been derived from BRAFi and MEKi treatments, melanoma persisters surviving chemotherapy and immunotherapy have also been characterized, though these studies are less common. One key difference in melanoma cells treated with chemotherapeutics vs targeted therapeutics is the induction of a senescence-associated secretory phenotype (SASP)^47^. The SASP is induced by DNA-damaging agents but not BRAFi and MEKi treatments^47^. In melanoma immunotherapy, RIG-1 activators are used to improve T cell activity, and this treatment strategy was shown to have similar effects on melanoma cells as targeted therapeutics^48^. In fact, RIG-1 activation led to dedifferentiated persister formation, with low MITF and high NGFR expression, among other effects^48^. However, it is unclear whether these persisters play the same role under immunotherapy vs other treatment strategies, as the dedifferentiated persisters were still vulnerable to CD8 tumor-infiltrating lymphocytes^48^.

### Metabolic alterations in melanoma persisters

Persisters of various cancer types surviving various treatments, including melanoma persisters treated with BRAFi and MEKi, frequently exhibit increased rates of fatty acid oxidation (FAO) (Fig. 1c)^38^. Reactive oxygen species (ROS) are also enriched in these persisters, and cycling persister formation is enhanced by antioxidant treatments^38^. Interestingly, the ability of surviving persisters to grow during treatment is also affected by FAO and ROS, as cycling persisters have higher FAO scores while controlling ROS levels more efficiently than the more abundant non-cycling persisters^38^. Similarly, *NRAS*-mutant melanoma cells surviving MEKi treatment exhibit high ROS and increased vulnerability to oxidative stress^42^. These cells also have an overall reduction in metabolic activity, with downregulated pentose phosphate pathway (PPP), explaining their vulnerability to oxidative stress^42^.

This redox-imbalanced state aligns with earlier observations identifying defective antioxidant defense as a fundamental feature of persister biology. Hangauer and coworkers demonstrated that persister cells broadly downregulate antioxidant programs and become highly dependent on glutathione peroxidase 4 (GPX4) to prevent lethal lipid peroxidation^12^, a vulnerability later corroborated in a preprint study^49^. These findings align with the phenotype commonly referred to as “iron addiction,” whereby increased dependence on iron-driven metabolic processes, together with compromised antioxidant defenses, renders cells highly vulnerable to ferroptosis^29^. Further, GPX4 has been identified as a vulnerability in *BRAF* V600E lung cancer persisters treated with BRAFi and MEKi^50^, demonstrating that responses to similar treatment strategies can be conserved across cancer types with consistent mutations.

Another common metabolic alteration in melanoma persisters is increased mitochondrial oxidative phosphorylation (OXPHOS) (Fig. 1c and Box 1). As FAO feeds OXPHOS^51^ and produces ROS^52^, it is rational to observe these changes together. In fact, OXPHOS was shown to be upregulated in melanoma persisters derived from BRAFi and MEKi treatments, and this alteration was supported by increased peroxisomal FAO via ACOX1 expression^53^. Additionally, by analyzing spatial changes in gene expression, spatially distributed persister populations with increased OXPHOS were observed within melanoma tumors^54^. High levels of OXPHOS were also observed in melanoma persisters with high expression of KDM5B^40^, the same chromatin modifier previously discussed^35^. In this case, these characteristics were consistent in persisters surviving single treatments with either BRAFi or cisplatin, a traditional chemotherapeutic^40^. Our research group has also shown increased utilization of mitochondrial metabolites in melanoma persisters surviving numerous traditional chemotherapeutics^33^, and untargeted proteomics in our follow-up study further revealed significant upregulation of mitochondrial protein abundance during treatment^55^, supporting enhanced mitochondrial metabolic rewiring. Mitochondrial oxidative respiration is also important to cancer persisters in general, as it is frequently found to be active in many types of cancer treated with various drugs, including traditional chemotherapeutics and targeted therapeutics^27^.

Additional metabolic alterations observed include increased lactate consumption rates in BRAFi persisters^44^ and increased UDP-glucose ceramide glycosyltransferase (UGCG) activity in persisters surviving BRAFi and MEKi^56^. We found that the BRAFi persisters also had increased survivability in minimal media^44^. Interestingly, the persisters with increased UGCG activity were also dependent on peroxisomal lipid metabolism^56^, which was previously mentioned^53^, further demonstrating the interrelation of many metabolic alterations in melanoma persisters.

Box 1 OXPHOS in cancer persisters: an evolutionarily conserved mechanism or not? Defining persister or drug-tolerant cancer cells as simply “dormant” misses the mark. It reduces a complex and dynamic biological state to a static label and overlooks the increasing body of evidence showing that these cells are not necessarily metabolically dormant. In fact, one of the most striking features of persister cells is their ability to rewire their metabolism in response to stress or therapy, resulting in a metabolic state that is not seen in actively growing cancer cells. Since Otto Warburg’s early observations nearly a century ago^126–128^, we know that most cancer cells can have increased glycolysis and reduced OXPHOS, even in the presence of oxygen. This metabolic trait is known as aerobic glycolysis. This metabolic strategy not only produces the energy required for survival but also generates the building blocks (e.g., nucleotides, amino acids, and lipids) for macromolecule biosynthesis required for the rapid growth of cancer cells^129,130^. Due to their reduced or absent proliferation, rather than funneling resources into anabolic pathways, persister cells potentially redirect their metabolic fluxes toward OXPHOS, a more efficient way to generate energy. Whether this is the unavoidable consequence of the growth arrest or mediated by an active and adaptive switch, this metabolic state is highly critical for the cells under stress, or in nutrient-poor and drug-loaded environments, because OXPHOS produces about 15 to 16 times more ATP than glycolysis^131^. This potentially helps cells like persisters produce energy fast and with fewer resources. What’s particularly fascinating is that this metabolic state is not unique to cancer persisters. We see a similar pattern in stem cells, microbial persisters, and even in senescent or normal cells that enter a tightly controlled non-proliferative state^132–136^. The similarities between these various cell types suggest that metabolic reprogramming isn’t just an anomaly of cancer but could be part of a broader evolutionary strategy. Persisters may be using this evolutionarily conserved program to alter themselves phenotypically, buy time to survive, maintain homeostasis, and possibly generate the energy needed for repair.

### Known anti-persister strategies

As many studies have found increased ROS levels and altered antioxidant pathways in melanoma persisters, several strategies have been developed using these characteristics as targets (Table 1). Specifically, the GPX4 inhibitors RSL3 and ML210 were identified to selectively kill melanoma persisters derived with BRAFi and MEKi treatments^12^. These chemicals induced ferroptosis in the persisters^12^, making ferroptosis an interesting target for melanoma persisters and cancer persisters in general. Interestingly, a preprint article has also linked the GPX4-dependence in melanoma persisters to ion channel dysregulation and depleted intracellular calcium^49^. Furthermore, GPX2 and other antioxidant genes were upregulated in cycling melanoma persisters, and erastin, a ferroptosis activator, was used to antagonize persisters^38^. The inhibition of bromodomain and extraterminal domain (BET) proteins is another interesting candidate for targeting melanoma persisters; the BET inhibitor (BETi) NEO2734 was shown to induce ROS and kill persisters^37^, by reducing the expression of antioxidant genes, including GPX2. *NRAS*-mutant melanoma MEKi persisters were also shown to be killed by ROS induction using the chemical neocuproine^42^.

**Table 1:** Therapeutic strategies for persisters

Class^a^	Sub-class	Drugs
*Redox/ferroptosis targeting*		
Highly relevant in melanoma persisters due to ferroptosis vulnerability	GPX4 inhibitors	RSL3 and ML210
	Ferroptosis inducer	Erastin
	ROS inducer	Neocuproine
*MAPK/RTK axis suppression*		
Critically relevant in melanoma persister cells, as they utilize the RTK–MAPK axis to adapt to MAPK inhibition therapy	RTK inhibitors	Lapatinib, poziotinib, infigratinib, and R428
	SHP2|pan-RAF inhibitors	SHP099, LY3009120 and RMC-4550
	ERK inhibitor	SCH772984
	SOS1 inhibitors	RMC-0331 and BI-3406
*Metabolic interventions*		
Highly relevant in melanoma persister cells due to their ability to shift metabolic pathways from glycolysis to oxidative phosphorylation, along with increased fatty acid oxidation, to survive treatment	ATP synthase|complex I inhibitor	Oligomycin
	Peroxisomal FAO and mitochondrial inhibitors	NNC 55-0396 and D, and L-threo PPMP and phenothiazine drugs (e.g., thioridazine)
*Transcriptional and translational disruption*		
Highly relevant since persisters are characterized by a non-genetic, reversible phenotype	BET inhibitor	NEO2734
	eIF4A inhibitor	Silvestrol
*Targeting senescence-related survival pathways*		
Highly relevant due to the senescence-like state, which is often induced by targeted therapies and chemotherapies	Senolytics	Piperlongumine
	Bcl2|Bcl-XL inhibitors	ABT-263, A-115, and ABT-199
*Interference with persister-specific signaling*		
Highly relevant, as it directly targets the non-genetic, drug-tolerant cell subpopulations	AhR|SMAD3 inhibitors	Resveratrol and SIS3
	FAK inhibitors	PF-562271 and defactinib
	KDM5B inducer | DHFR inhibitor	Cpd1 | TMECG

^a^Experimental approaches in melanoma include redox/ferroptosis targeting (GPX4 inhibition, ROS induction), MAPK/RTK axis suppression (RTK, SHP2, RAF, ERK inhibition), metabolic interventions (ETC, FAO, UGCG inhibition), transcriptional and translational disruption (BET, eIF4A inhibition), targeting senescence-related survival pathways (senolytics, BCL-2/BCL-XL inhibition), and interference with persister-specific signaling (AhR, SMAD3, FAK inhibition). Many of these have been tested in melanoma broadly, while others directly address persister vulnerabilities. See the text for details.

As BRAFi and MEKi treatments target the MAPK/ERK pathway, evasion of these treatments tends to involve reactivation of the pathway^31,32^. Therefore, some strategies to eliminate these persisters target proteins upstream or downstream from BRAF and MEK to further suppress the pathway. Due to the receptor-driven rewiring of MAPK signaling in melanoma persisters surviving BRAFi and MEKi, co-treatment with RTK, SHP2, or pan-RAF inhibitors suppressed pulsatile ERK activity and prevented drug adaptation^31^. SHP099 and LY3009120 were used as SHP2 and pan-RAF inhibitors, respectively, while lapatinib, poziotinib, infigratinib, and R428 were used as RTK inhibitors^31^. Similarly, the SHP2 inhibitor RMC-4550, as well as the SOS1 inhibitors RMC-0331 and BI-3406, were used to prevent ERK reactivation in BRAFi and MEKi persisters^32^. In the same study, ERK was also directly inhibited with SCH772984^32^. ERK inhibition is indeed a compelling strategy to eliminate BRAFi and MEKi persisters, and several ERK inhibitors are currently under clinical investigation^57^. Developing novel strategies to further suppress the MAPK/ERK pathway will continue to improve clinical outcomes for *BRAF*-mutant melanoma treatment; however, as we have observed with combined BRAFi and MEKi treatments, persisters may continue to rewire their proliferative signals to evade any new drugs that ultimately target the same pathway.

Targeting key metabolic processes in melanoma persisters is another interesting strategy that has been demonstrated in numerous studies. Due to the importance of mitochondrial respiration in melanoma persisters, oligomycin, which inhibits ATP synthase, as well as complex I inhibitors, reduced the survival rates of melanoma BRAFi persisters^40^. Interestingly, we have also shown that electron transport chain (ETC) inhibitors reduce survival of melanoma persisters exposed to traditional chemotherapeutic treatment^33^. Melanoma persisters were also sensitized to BRAFi and MEKi treatment by inhibiting peroxisomal FAO, which was achieved by thioridazine treatment, producing similar results to ACOX1 knockdown^53^. Similarly, inhibition of peroxisome biogenesis and UGCG-dependent ceramide metabolism with NNC 55-0396 and D,L-threo-PPMP, respectively, induced apoptosis in BRAFi and MEKi persisters^56^.

Several anti-persister strategies that target persister drug adaptation have also been shown. For example, mRNA translation remodeling was prevented in BRAFi and MEKi persisters using an eIF4A inhibitor, silvestrol, leading to enhanced drug efficacy^36^. Also, due to the FAK-dependent AKT activation in BRAFi and MEKi persisters, FAK inhibition with PF-562271 and defactinib prevented persister survival^43^. Interestingly, in this case, FAK inhibition only allowed melanoma cells with genetic resistance mechanisms to survive treatment, leading to more predictable resistance mechanisms and the absence of multi-drug resistance^43^. Similarly, in another study, inhibitors of FAK, JNK, and Src reduced NGFR expression and sensitized cells to BRAFi and MEKi treatment^34^. The authors also found that BETi treatments suppressed NGFR and enhanced drug efficacy^34^, linking the epigenetic adaptations of persisters to the previously discussed ROS vulnerability^37^. Other strategies preventing drug adaptation include the inhibition of AhR and SMAD3 with resveratrol^46^ and SIS3^41^, respectively, leading to increased BRAFi and MEKi sensitivity.

Additional ideas for targeting melanoma persisters include using senolytics^47^ as well as limiting tumor plasticity with “persister state-directed transitioning”^35^. Senolytics, including Bcl2 and Bcl-XL inhibitors as well as piperlongumine, killed senescent-like persisters surviving treatment with traditional chemotherapeutics^47^. The inhibitors of Bcl2 and Bcl-XL, which were used, include ABT-263, A-115, and ABT-199^47^. This strategy is more relevant for enhancing treatment with DNA-damaging chemotherapeutics than with targeted therapeutics, as the chemotherapeutic treatments generally lead to SASP-induction^47^. The “persister state-directed transitioning” strategy is unique, as it aims to lock all the treated cells in a persister state and capitalize on the vulnerabilities of the persister state^35^. This strategy was demonstrated via co-treatment with 2-phenoxyethyl 4-(2-fluorophenyl)-2,7,7-trimethyl-5-oxo-1,4,5,6,7,8-hexa-hydro-quinoline-3-carboxylate (Cpd1) and 3-O-(3,4,5-trimethoxybenzoyl)-(-)-epicatechin (TMECG)^35^. In this study, Cpd1 was used to induce KDM5B expression, promoting a persister state with high melanocytic differentiation, and these persisters were accordingly shown to be vulnerable to TMECG, a prodrug that is processed by tyrosinase^35^.

## Future directions: what is missing and what must be done?

**Fig. 2:**
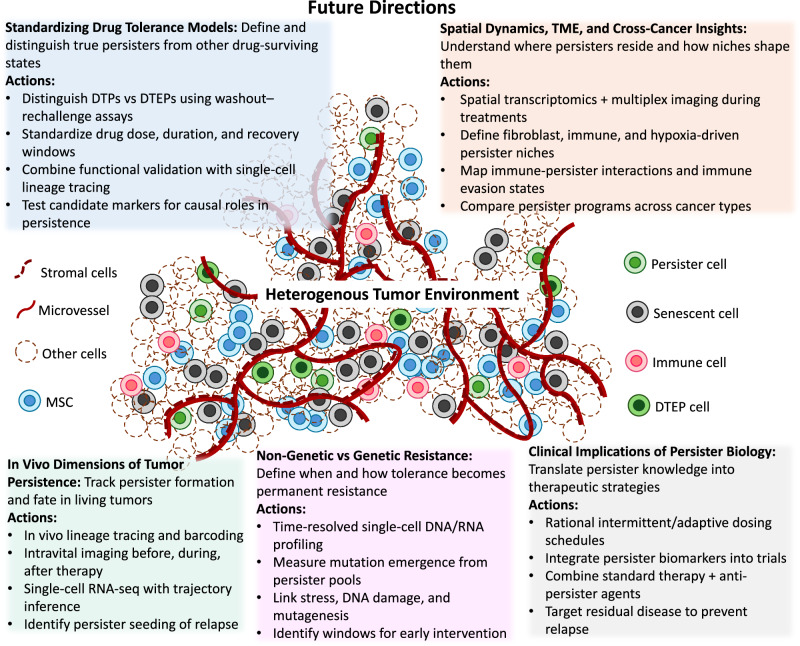
Future directions. Key priorities include standardizing experimental models to distinguish drug-tolerant persisters from other cell types [e.g., melanoma stem (MSC) and senescent cells], extending in vivo studies to account for microenvironmental effects, spatial heterogeneity, and immune interactions, and clarifying the interplay between genetic and non-genetic mechanisms of resistance across cancers, as well as the clinical implications of persisters.

While studies in vitro have shed light on some aspects of persister biology, we still know surprisingly little about how these cells behave in living systems or across different melanoma subtypes. Defining their identity and vulnerabilities in vivo remains a major gap. Bridging this gap requires more consistent experimental models, better use of single-cell and spatial profiling technologies, and a shift toward studying persisters in settings that more closely reflect the tumor microenvironment (Fig. 2). This section outlines critical directions for future research: what’s missing, and what needs to happen next to target these elusive cells and reduce the risk of melanoma relapse.

### Standardizing drug tolerance models in melanoma

The field needs clear, consistent methods to find, isolate, and measure drug-tolerant persister cells, because the way persisters are defined and studied has a direct impact on the interpretation of experimental results. In practice, two main strategies are used, depending on treatment duration and frequency, and they can yield very different outcomes. Short-term high-dose treatments, typically lasting two to four days, induce a transient tolerance state^11,12,53^. These cells, often referred to as drug-tolerant persisters (DTPs) and analogous to bacterial persisters, survive temporarily under therapy but quickly regain sensitivity and proliferative capacity once the drug is removed^11^. In contrast, prolonged exposure to therapy, often for several weeks to months, tends to generate more stable populations known as drug-tolerant expanded persisters (DTEPs, Fig. 1a)^11,28^. Conceptually, DTPs and DTEPs may represent a continuum, in which early, reversible persister states serve as a reservoir from which DTEP populations emerge and form slow-growing colonies under continued drug exposure^11^. Although DTEPs exhibit proliferative capacity during treatment, early DTEPs remain largely non-genetic and reversible, given that drug withdrawal followed by extended drug-free passaging can restore parental-like growth behavior and drug sensitivity^11^. This proliferative capacity in the absence of resistance-conferring mutations is thought to reflect stabilization of adaptive non-genetic programs, including persistent epigenetic remodeling, transcriptional and translational rewiring, and metabolic adaptation^11,38,58,59^, which together enable limited growth without reactivation of full oncogenic signaling. Over time, however, prolonged persistence under therapeutic pressure may increase the likelihood of genetic diversification and eventual acquisition of stable resistance mechanisms^13^, although the timing and molecular determinants of this transition remain to be further explored.

Studies in melanoma and lung cancer have shown that extended inhibition of targets like BRAF or EGFR can select for such slow-growing, drug-adapted clones^11,60^; therefore, distinguishing DTPs from DTEPs is necessary and requires functional validation. Reversibility should be demonstrated using washout—rechallenge assays, in which drug-surviving cells recover in drug-free conditions before re-exposure to therapy. Rapid restoration of proliferation and drug sensitivity supports a DTP-like state, whereas sustained tolerance after short recovery periods is more consistent with a DTEP-like state. Because DTEPs can regain sensitivity only after prolonged recovery, explicit reporting of recovery duration, rechallenge conditions, and growth behavior under continuous drug exposure is essential for experimentally separating transient persistence from expanded, yet still reversible, drug tolerance.

While highly useful, these approaches are complicated by the high degree of heterogeneity that either preexists or is induced by the therapy itself. Drug-treated melanoma cultures do not just contain persisters; they also include dying cells, pre-resistant clones, and quiescent, stem-like populations^39,61–63^. This complexity makes it hard to confidently attribute observed phenotypes specifically to persisters. Without consistent definitions around timing, drug dosing, and lineage-tracing methods, there’s a high probability of lumping together biologically distinct cell types under the same label. To move the field forward, techniques like single-cell lineage tracing, paired with live-cell imaging and molecular barcoding^38,64–66^, might be used to track individual cells over time and clarify their fates after treatment (although this is labor-intensive). While persistence is generally confirmed through functional washout-rechallenge models, any candidate markers must be tested for their actual role in persistence, rather than assumed based on correlation alone. These steps are critical for separating persister-specific biology from broader, nonspecific responses to drug stress.

As noted above, the heterogeneous nature of drug-treated cell populations becomes increasingly complex in the presence of additional phenotypic variants, such as melanoma stem cells (MSCs) and senescent cells. Distinguishing among these subpopulations is challenging, as their phenotypic features often overlap and may shift dynamically between states (see Box 2, and Table 2). Traditionally, stem-like cells are defined by their capacity to initiate tumors in experimental models, but this property is not always exclusive to them, given that similar behaviors may also be seen in persister cells or in cells exposed to SASP signaling. Even the markers we often rely on, like CD271 or ALDH, can be found in more than one cell type, depending on the conditions^12,33,53^. In the long run, separating these cell states will require studying them from multiple angles: how they behave over time, how they function, and what defines them at a molecular level. This integrated view is what we will need to design smarter therapies, ones that not only target specific populations but also block the transitions between different phenotypic variants that make melanoma so hard to treat in the first place. To distinguish these cell types, we need to bring in more advanced tools. Technologies like single-cell RNA sequencing, combined with epigenetic profiling and metabolic imaging, can help track how these cells change over time and under stress. Using reporter systems for stem cell biomarkers can help identify MSCs^67,68^; reporters for cyclin-dependent kinases, DNA damage response, SA-β-gal activity, and SASP factors can help distinguish senescent cells^69,70^; and proliferation markers may help track persister cells, which are known to rapidly transition from persistence to proliferative state^71,72^.

**Table 2:** MSC, senescent and persister cells: similarities and differences

Feature	Melanoma stem cells (MSCs)^a^	Melanoma senescent cells^a^	Melanoma persister cells^b^
Proliferation	Slow-cycling or self-renewing	Irreversibly arrested, except in rare cases of forced epigenetic reprogramming	Transiently non-dividing or slow-cycling
Reversibility	Yes, can differentiate or remain quiescent	Generally, no; irreversible arrest (though some escape in cancer)	Yes, re-enter the cell cycle after drug removal
Origin	Developmental plasticity, EMT, dedifferentiation	DNA damage, oncogene activation, chemotherapy	Drug-induced stress, stochastic or adaptive
Markers	CD133, ABCB5, CD271, ALDH, Sox10	p16, p21, SA-β-gal, γH2AX	MITF^low^, NGFR^high^, FAO genes
Metabolic state	Adaptable; often ALDH⁺, oxidative stress-resistant	High metabolic and secretory (via SASP)	Oxidative phosphorylation, FAO-dependent, ROS-tolerant
Drug tolerance/resistance	High tolerance due to the slow growth, or via efflux pumps (e.g., ABCB5) and ALDH; not genetically resistant	High tolerance due to growth arrest; promotes tolerance in neighbors (SASP); not genetically resistant	High tolerance due to the slow growth or growth arrest promotes resistance; it is not genetically resistant.
Immune interaction	Immune evasive; low MHC-I, high PD-L1; enriched in niches with limited immune surveillance.	Active immune signaling via SASP recruits inflammatory cells, but can promote immune escape	Potentially hide from immune attack in niches (requiring further studies)
Plasticity / EMT	Highly plastic; undergo EMT and phenotype switching	Limited plasticity; fixed arrest, though dedifferentiation is possible	Highly plastic; often transitions into an EMT-like state
Tumorigenic potential	High; initiate and sustain tumor growth	Low individually, but contribute to relapse via niche alteration	Can lead to relapse by seeding resistant outgrowth
Role in recurrence	Major source of recurrence/metastasis	Promote recurrence through niche remodeling and inflammation	Seed for relapse after therapy removal
Contribution to Vasculogenic Mimicry (VM)	Yes, participate in VM (VEGF, CD144⁺)	No direct role; may influence angiogenesis via SASP	Indirect via niche factors; not directly VM-forming (requiring further studies)
Functional criteria distinguishing these cell states	Tumor-initiating capacity in xenografts seeded with few cells; long-term self-renewal; lineage stability across passages; and existence of stem cell biomarkers	Sustained and largely irreversible growth arrest confirmed by persistent DNA damage response and SASP despite drug withdrawal	Survival under acute drug exposure followed by reversible recovery of proliferation and drug sensitivity upon drug washout

^a^Stem and senescent cells in melanoma are outside the scope of this review. The information provided here reflects general knowledge in the field and is intended only to differentiate these cell types from persisters. For detailed discussions and references, please refer to the following review articles^120–125^.

^b^For detailed references about persisters, please see the previous sections of this article.

Box 2 Cancer stem, persister, and senescent cells in melanoma: convergent or distinct? Metabolic rewiring, growth arrest state, or dormancy represents a physiological state of a cell that can be shared by various phenotypic variants such as melanoma stem cells (MSCs), senescent cells, and persister cells. Although we are not planning to review MSCs and senescent cells here (as they are out of our scope), we would like to highlight that these three different cell types often play similar roles in helping cancer resist treatment and facilitate recurrence (see Table 2, highlighting the similarities and differences between these variants). MSCs are relatively stable variants and carry stem cell biomarkers such as CD133, ABCB5, CD271, and increased aldehyde dehydrogenase (ALDH) activity^120–122^. They have the ability to renew themselves, shift their identity, and initiate new tumor growth. These cells tend to live in protected niches that are rich in blood vessels and shielded from immune attack, where they also contribute to forming vein-like structures through vasculogenic mimicry^120^. Melanoma persister cells are different from MSCs. They are not necessarily preexisting cell types, as they can emerge in response to treatment. They grow slowly (or not at all), tolerate drugs, and switch on specific gene programs (e.g., low MITF expression and high levels of NGFR)^15,34,43,44^. Metabolically, they shift away from growth-focused pathways and rely more on oxidative phosphorylation and fatty acid metabolism. These cells don’t have consistent surface markers, but what makes them particularly interesting is their ability to transition into a fast-proliferative state once the treatment pressure is gone, unlike MSCs, which tend to be less proliferative regardless of drug presence (unless they differentiate into more actively dividing cells). In contrast, senescent cells, in general, enter an irreversible growth arrest in response to stresses like DNA damage or activation of oncogenes^124,125^. They continue to release a mix of inflammatory signals via the SASP, which can promote tumor growth, drive EMT, and alter the immune response in the tumor’s favor^124,125^. Unlike MSCs and persisters, senescent cells usually lack the flexibility to switch identities or re-enter the cell cycle, but they still play a powerful role in shaping the tumor environment. Though they differ in origin and behavior, all three cell types share core traits; they are non-growing or slow-growing and manage to survive in harsh environments. These shared features make them major contributors to minimal residual disease and relapse in melanoma and important targets for new therapeutic strategies.

### Understanding spatial dynamics, tumor microenvironment, and cross-cancer insights

There are a few new areas in melanoma persister research which we should give additional attention to in the future. For example, persistence appears to be a dynamic phenomenon not only temporally, but also spatially^54^. In a recent article, combined image analysis of melanoma tumor samples from mouse xenografts and spatial transcriptomics were used to study spatial changes of gene expression during treatment^54^. The spatial effects on drug tolerance have not been discussed in other melanoma persister research articles; these effects need to be further described to give us further understanding of persister dynamics within tumors themselves.

Closely linked to spatial organization is the tumor microenvironment (TME), which plays an essential and active role in shaping melanoma cell responses to therapy and in stabilizing drug-tolerant persister states. Rather than acting as a parallel or secondary layer of resistance, microenvironmental cues continuously interact with intracellular signaling, metabolic, and epigenetic programs within melanoma cells, potentially facilitating reversible survival states during treatment. This integrative view is increasingly supported by both melanoma-focused studies and broader analyses of drug-tolerant persister biology across cancer types^73–76^. Cancer-associated fibroblasts, and more specifically melanoma-associated fibroblasts (MAFs), represent key regulators of this adaptive niche. MAFs are dynamically activated by melanoma-derived signals and therapy-induced stress and, in turn, secrete cytokines and growth factors such as IL-6, IL-8, LIF, and TGF-β^75^. These signals promote melanoma cell survival, phenotypic plasticity, and invasive behavior while reinforcing intracellular rewiring programs associated with drug tolerance^75^, including modulation of MAPK signaling, activation of YAP/TAZ pathways, and induction of stress-response transcriptional states^77–82^. Importantly, MAF-derived signaling is amplified under hypoxic conditions^83^, underscoring that stromal cues and physicochemical stressors act synergistically rather than independently. However, how these microenvironmental signals are sensed, integrated, and translated into specific persister-associated states at the cellular level remains poorly defined.

In parallel, chronic antigen exposure and therapy-induced inflammation can drive immune exhaustion, particularly in cytotoxic CD8⁺ T cells, reducing immune-mediated clearance of residual tumor cells^84–86^. Macrophages and other myeloid populations further contribute to immunosuppression^87,88^, and MAF-driven polarization toward immunosuppressive phenotypes enhances IL-10 production and dampens cytotoxic responses^89^, potentially creating a permissive niche for persister survival. In this context, persister cells may not be passive survivors but may actively adopt transcriptional and epigenetic states promoting immune evasion, a feature that can be observed across multiple tumor types but not yet fully established for persister populations.

Although melanoma is highly immunogenic, interactions between melanoma cells and the immune system can contribute to drug tolerance and the acquisition of drug resistance^90^. Specifically, Ccr2+ monocyte-derived macrophages were shown to infiltrate the TME at the time of persister formation during treatment with BRAFi and MEKi^90^. Further, TME Ccr2 proficiency or deficiency influenced the development of stable or unstable resistance, respectively^90^. In addition to studying the effects of immune cells themselves, more work should be done to characterize responses of melanoma cells and melanoma tumors to relevant immunotherapy methods. For example, RIG-1 activation induced a melanoma persister state, which was still vulnerable to immune cells^48^. We still do not know the role of melanoma persisters in response to other immunotherapy methods.

In addition to immune and stromal interactions, physicochemical stressors within the TME may directly reprogram persister cell physiology. Hypoxia and extracellular acidosis are defining features of treated melanoma lesions, arising from aberrant vasculature, high metabolic demand, and therapy-induced vascular disruption^74,91–93^. These conditions necessitate adaptive metabolic rewiring to maintain redox balance, ATP homeostasis, and intracellular pH^93,94^. Hypoxia-driven transcriptional programs and acidosis induce drug-tolerance states^95,96^ that closely resemble persistence. Notably, hypoxia and acidity also enhance extracellular vesicle release, facilitating intercellular communication that propagates adaptive traits across tumor and stromal compartments^97–101^. Metabolic constraints imposed by hypoxia and nutrient limitation may intersect with transcriptional plasticity, epigenetic memory, and stress-response pathways implicated in persister biology. Rather than reflecting fixed, cell-intrinsic programs, persister states might be maintained through dynamic, bidirectional interactions between tumor cells and their microenvironment. In melanoma, such interactions may stabilize low-proliferative or dormant-like states that enable survival during therapy; however, how microenvironment-driven metabolic and stress cues quantitatively shape persister frequency, longevity, and resuscitation remains unresolved.

We should also prioritize research on the connections and similarities between melanoma persisters and other types of cancer persisters. One such similarity, for example, is the vulnerability to GPX4 inhibition; this is not only relevant in melanoma BRAFi and MEKi persisters but also *BRAF*-mutant lung cancer persisters^50^. Any novel, clinically relevant anti-persister strategies that are broadly applicable to several cancer types would prove to be invaluable.

### In vivo dimensions of tumor persistence

As highlighted above, the conditions inside a tumor are far more complex in vivo than in vitro. Cells in vivo are constantly navigating a chaotic microenvironment, given that oxygen levels, nutrients, immune cells, pH levels, and signaling cues always shift over time and space^102–105^. These stressors do not just passively affect the tumor; they actively shape which cells survive, adapt, or die; therefore, it is very critical to understand the impacts of these stress factors on persistence. Tumor cells are highly heterogeneous in vivo, and they differ in their gene expression, metabolism, proliferation rate, and interactions with the surrounding microenvironment^106–108^. This diversity means that some cells are more likely to become persisters than others, depending on where they are in the tumor and what signals they’re receiving. Understanding how these local “niches” influence persister formation will be critical for pinpointing vulnerabilities we can actually target. To move the field forward, we need to bring all these dimensions together (spatial context, lineage dynamics, immune interactions) and study them in vivo, where the biology is most authentic. Only by mapping how persisters form, survive, and interact with their environment in real tumors can we develop therapies that prevent relapse at its root. As mentioned above, techniques like lineage tracing and single-cell barcoding allow us to follow the fate of individual cells over time, while single-cell RNA-seq combined with trajectory analysis can map how cells change at the transcriptional level as therapy unfolds^109,110^. Intravital imaging lets us monitor tumor cells in real time within their native environment before, during, and after drug treatment^111^. Although challenging, a multi-pronged approach that integrates all these techniques with in vivo models will be essential for advancing our understanding of persister cells.

### Non-genetic resistance mechanisms vs. genetic evolution in melanoma persisters

Unfortunately, treatment pressure doesn’t just help persister cells form; it gives them room to evolve. In melanoma, persistence during BRAF/MEK inhibition often begins with early, reversible adaptations. These changes are not permanent, but they are enough to buy time for cells to acquire the mutations that eventually make them genetically resistant. It is highly expected that therapy-induced stress accelerates mutation rates, or that compromised DNA repair leaves the door open to genetic change^13,39,61^. What is still unclear is when and how this shift happens. The transition from a drug-tolerant, plastic state to a genetically resistant one is difficult to track, especially in vivo. Real tumors are dynamic ecosystems; constant changes in tumor environments, metabolic stress, and toxic products definitely shape the trajectory of a persister cell and resistance development^112,113^, but the exact mechanisms remain unclear. To answer these questions, we’ll need better tools, such as single-cell genomic profiling for detecting mutations and their formation timing, and long-term in vivo models that can capture these transitions as they unfold. Clinically, this early window, before resistance is genetically hardwired, could be our best shot at intervention. But to take advantage of it, we need a deeper understanding of how environmental stress, plasticity, and mutagenesis interact in the real context of a tumor.

### Clinical implications of persister biology

Although drug-tolerant persister cells are now well established in experimental melanoma models, their direct targeting in the clinic remains challenging due to their transient, non-genetic, and phenotypically heterogeneous nature. Consequently, to date, no melanoma clinical trials have been explicitly designed with persister cells as a formal therapeutic endpoint. Nevertheless, several clinical strategies currently under investigation can be conceptually aligned with persister biology and implicitly aim to exploit key features of persistence, including reversibility, the link between persistence and the emergence of stable resistance, and cancer recurrence.

A prominent example of a clinically relevant strategy conceptually aligned with persister biology is intermittent treatment, in which therapy is administered in predefined on–off cycles rather than continuously. From a mechanistic perspective, intermittent treatment strategies have been used in studies of bacterial persisters^114,115^. In these systems, bacterial persisters occupy low-proliferative, drug-tolerant states during treatment, while drug withdrawal permits a fraction of cells to exit these protected states and transiently re-enter drug-susceptible physiological programs, enabling their elimination upon subsequent drug re-exposure. Similar principles may apply to cancer cells, where reversible drug-tolerant cells can exit persistence following drug removal.

In melanoma, the intermittent treatment has already been explored with BRAF and MEK inhibitors in clinical trials (e.g., NCT02583516-completed, NCT03352947-completed, and NCT02196181-active, not recruiting), with the rationale that treatment breaks might alter tumor cell fitness or drug response dynamics. Unfortunately, the published outcomes of these trials did not demonstrate improved progression-free or overall survival with intermittent dosing relative to standard continuous treatment^116,117^. However, mathematical models of bacterial persisters showed that the timing and ratio of drug on–off cycles are critical determinants of persister eradication, and that non-optimal scheduling can fail to eliminate persisters or even promote their survival^114,115^. A key future direction will be the rational design and testing of dosing regimens that explicitly account for the formation, maintenance, and exit of drug-tolerant cancer cells, which may enable more mechanistic interpretation of clinical outcomes and improve the translational relevance of intermittent or adaptive treatment strategies.

More recent clinical efforts address a distinct but related problem: acquired resistance. For example, ctDNA-guided adaptive BRAF/MEK inhibitor therapy (e.g., NCT06470880, active but not yet recruiting) is designed to detect emerging molecular resistance and adjust treatment accordingly. While not aimed at persister states, such resistance-focused adaptive strategies underscore a broader shift toward dynamic treatment modulation in response to tumor evolution. An important future direction will be to determine whether integrating persistence-related biomarkers or scheduling principles into such adaptive frameworks can improve long-term disease control without compromising clinical feasibility.

Beyond treatment scheduling, several completed clinical trials have aimed to prevent melanoma recurrence after apparent disease clearance, an objective conceptually related to limiting the survival or outgrowth of residual, therapy-tolerant persister cells. These include vaccine-based and immune-stimulatory approaches evaluated in the adjuvant setting, such as booster peptide vaccination in previously vaccinated patients with resected melanoma (NCT01989559, completed) and a randomized phase III trial testing sargramostim (GM-CSF), peptide vaccination, or their combination following surgical resection of high-risk disease (NCT01989572, completed). Although immunogenic personalized neoantigen vaccines elicit measurable antigen-specific T-cell responses and, in some cases, reduce disease recurrence^118^, vaccine-based therapies have not consistently yielded improvements in relapse-free or overall survival across study populations^119^. From a persister perspective, such results raise the possibility that residual melanoma cells may occupy protected, slow-cycling, or immune-evasive states that are insufficiently targeted by current adjuvant strategies. Future approaches may therefore require more precise integration of immune reinforcement with an improved understanding of the physiological states that allow residual tumor cells to persist and later re-emerge.

## Conclusion

As outlined in this review, a wealth of research has been performed to develop new targets and treatment strategies to address melanoma persisters. However, none of these treatment strategies has been tested clinically. Clearly, more research needs to be done to clarify the characteristics and vulnerabilities of melanoma persisters and find novel methods to eradicate them in the clinical setting. Minimizing the effects of persisters will eventually lead to enhanced treatment methods; this is especially true for metastatic melanoma, given the high persister levels and high rates of recurrence for this disease.

### Reporting summary

Further information on research design is available in the Nature Portfolio Reporting Summary linked to this article.

## Supplementary information

Reporting Summary
